# Factors influencing general practitioners in the referral of elderly cancer patients

**DOI:** 10.1186/1471-2407-11-5

**Published:** 2011-01-06

**Authors:** Fleur Delva, Emilie Marien, Marianne Fonck, Muriel Rainfray, Jean-Louis Demeaux, Philippe Moreaud, Pierre Soubeyran, Annie J Sasco, Simone Mathoulin-Pélissier

**Affiliations:** 1 Institut Bergonié, Comprehensive Cancer Centre, 229 cours de l'Argonne, 33076 Bordeaux Cedex, France; 2 CHU Xavier Arnozan, Avenue du Haut-Lévêque, 33604 Pessac Cedex, France; 3 Université Victor Segalen, Bordeaux 2, 146 rue Léo Saignat, 33076 Bordeaux Cedex, France; 4 Union Régionale des Médecins Libéraux d'Aquitaine, 105 rue Belleville, 33074 Bordeaux Cedex, France; 5 Inserm U897, Equipe d'Epidémiologie pour la prévention des Cancers and CIC-EC 7, Université Victor Segalen Bordeaux 2, 146 rue Léo Saignat, 33076 Bordeaux, France

## Abstract

**Background:**

A number of studies have identified advanced age as a barrier to accessing specialised oncological care. Many factors can influence the care provided for elderly patients after a diagnosis of cancer has been established or is suspected. Only one European study has analysed the decision processes leading general practitioners (GPs) to refer elderly patients with cancer to oncologists. The objectives of the current study are to describe the factors that influence these decisions and to identify the particular factors and GP characteristics that are associated with systematic referral of these patients in South-West France.

**Methods:**

This is a cross-sectional study on a representative sample of GPs in Aquitaine, South-West France. Questionnaire items were selected using a Delphi consensus approach and sent by post. Two logistic regression models were constructed to investigate GPs' decisions to refer these patients.

**Results:**

The response rate obtained was 30%. Half of the general practitioners reported "always" referring their elderly cancer patients to oncologists. More than 75% reported being influenced by patient-related elements (patient and/or family wishes, comorbid factors, unsuitability of invasive investigations, physical and mental autonomy), by cancer-related elements (severity of symptoms, expected side-effects) and an organisational element (whether the general practitioner was used to collaborating with oncologists). Logistic regression analysis showed that cancer site and organisational difficulties in patient management were significantly associated with the decision to refer elderly patients with early-stage cancer. For advanced stages, oncology training, patient age, organisational difficulties in patient management and stage of cancer were significantly associated with the decision to refer elderly patients.

**Conclusions:**

Cancer-linked factors and organisational difficulties have been highlighted as influencing the decisions of GPs in the referral of elderly patients to a cancer team. These results highlight the need to implement continuous medical education specific for the management of elderly patients, to better apprehend the nature of these difficulties and to suggest solutions suited to local settings.

## Background

As cancer incidence increases with age, with 30% of cancers in 2005 occurring in patients over 75 years [1], the burden of cancer in the elderly is rising and has an impact on the cost and organization of care. Given the size of the elderly population and this increased incidence of cancers, it is important to optimise the way in which elderly cancer patients are cared for and their quality-of-life. Cancers are often diagnosed late in the elderly [2,3]. Potential explanations for this are, among others, distance from care facilities, loss of autonomy and the fact that the elderly tend to miss screening measures. The general practitioner (GP) has a central role in the organisation of care provision: it is s/he who decides where to refer the patient [4], and the care provided may vary in quality according to the facility selected for referral [5]. For elderly people, the GP's role is particularly important, since elderly patients are generally more dependent than younger patients [4,5]. Once the diagnosis of cancer is established in clinical practice, numerous factors can influence the decision as to how elderly cancer patients are cared for. These factors include age, acceptability of the treatment by the patient or the patient's family, mental status, the presence of disability, the facility to which the patient might be referred, and the attitude of the physician [6,7].

The main aim of this study was to describe the factors influencing GPs in South-West France (Aquitaine) in their decisions whether or not to refer elderly cancer patients to oncology teams, using a cross sectional design. The detailed objectives of the study were to: 1) estimate the proportion of GPs who refer their patients according to cancer stage (early or advanced), 2) identify factors associated with this decision among influential elements and individual GP characteristics, and 3) use two clinical case vignettes to identify the factors influencing GPs in clinical situations in their decision to refer an elderly patient presenting with cancer to specialised care.

## Methods

### Participants

One thousand five hundred GPs in private practice (note that GPs in France have self-employed status) were drawn from exhaustive listings held in regional healthcare professional databases of all GPs in the region (n = 4006) using systematic random sampling, stratified by the 5 regional administrative districts. Study data were collected using a questionnaire sent by post in December 2007, and two reminders were sent as needed in January and February 2008. The study was approved by the consultative committee on the processing of information in medical research of CNIL (French national commission on individual privacy).

### Materials and methods

To obtain explicit consensus on items to be included in the questionnaire covering elements likely to influence GPs in the referral of their elderly cancer patients to specialised cancer teams, we used the Delphi consensus method [8,9]. The questionnaire was constructed from items selected by the consensus method and from a questionnaire already used in a North-American study [10]. It was piloted on 10 GPs, and the wording was altered according to their comments. The final version comprises four sections and two clinical case vignettes. Section A concerns the GP's elderly patients overall (GP's perception of the age at which a person is "elderly", percentage of elderly people among patients overall). Section B concerns referral to a specialised oncology team for the GPs' elderly patients with cancer, in two categories: early or advanced cancer. This section included the key items in the questionnaire: percentage of patients referred to a cancer team and list of factors liable to influence GP decisions (developed using the Delphi consensus method). Section C explored any training received by the GPs in oncology or geriatrics (in France, GPs can graduate in geriatrics opposed to oncology where they can only train in an oncology unit during an internship), and the perception they had of the way elderly cancer patients are cared for in France. Section D recorded the characteristics of the responding GPs (number of years in practice, practice setting (urban or rural), weekly working hours, work situation (alone or in partnership). Finally, two clinical case vignettes were provided to investigate the GP's attitude in a clinical situation: one case of prostate cancer and one sigmoid colon cancer case. The questions covered the GP's attitude for deciding how the patients should be cared for and asked them the three most influential elements for the general referral decision regarding early or advanced elderly cancer patients (selected from a list identical to that used in Section B on general referral decisions). The questionnaire is displayed in an additional file (additional file 1).

### Analysis

To describe the participating physicians and their general attitudes, we used means, standard deviations, medians, range, percentages and frequencies. Inter-group comparisons were performed using the Chi^2 ^test, the Fisher's exact test, the Student's t-test or Wilcoxon's test according to the distribution. All tests were performed with alpha set at 5%. Regarding the referral decision (questionnaire section B), there were five possible responses: "very rarely (<10% of the time)", "rarely (10 to 25%)", "sometimes (25 to 50%)", "often (50 to 75%)" and "always (>75%)". These responses were then grouped into two categories: "does not always refer" (very rarely, rarely, sometimes, and often) and "always refers" ("always refers"). The proportion of GPs "always" referring their elderly cancer patients according to the stage of the disease (early or advanced) were then described using counts, percentages and 95% confidence intervals (CI).

For the two clinical case vignettes (questionnaire section E), the GPs' attitudes were described, and then the elements selected as influencing their decisions were compared against the more general results given in Section B of the questionnaire. Two logistic regression analyses were on the general decision of whether or not to refer an elderly patient according to the stage of the disease (questionnaire section B). For each model, the variable to be explained was the decision to refer a patient (or not) and the explicative variables were GP characteristics (age, gender, practice setting, numbers of year of medical practice, work situation, weekly work time, training in oncology, training in geriatrics) and factors influencing them in the referral or not of their patients (factors from section B liable to influence GP decisions such as patient age, anatomical localisation or presence of good clinical practice guidelines). The variables that were significant in the univariate regression analyses at p < 0.20 and also those reported in the literature, were introduced into the multivariate logistic regression models. For each model, explicative variables were removed using a stepwise descending selection procedure, as set out by Hosmer and Lemeshow [11]. This produced a model for each cancer stage (early and advanced), and model fit was ascertained using the Hosmer and Lemeshow goodness-of-fit test [11].

## Results

### General GP characteristics

Of the 1500 GPs approached in Aquitaine, 30% (436) had responded to the questionnaire after a maximum of two reminders. Among these GPs, 75% were men (321) (table 1) and the mean age was 50 years (sd: 8.9, range 28-70). On average, respondent GPs had been practising medicine for 21 years (sd: 9.4, range 1-40). The average weekly working time was 55 hours (sd: 11.9, range 10-90). Two thirds of the GPs (321) were working in an urban setting. More than half were working in partnerships. More than 90% of the GPs deemed that over the age of 70 a patient was to be considered to be "elderly". Around 50% reported having a proportion of more than 20% of elderly people over 70 among their patients overall. Among the GPs in the present study, 30% had received training in geriatrics and 15% in oncology. Nearly 65% of the GPs (243) considered that there were suitable courses available for training in the care of elderly cancer patients. The main suggestions proposed for the improvement of training courses of this sort related to continuing education and emphasised the need for training courses in their local area which were suited to their working hours.

**Table 1:** Characteristics of 436 general practitioners that responded to the questionnaire

	N	(%)
**Gender**		
Male	321	(73.6)
Female	115	(26.4)
**Age**		
<50 yrs	177	(40.6)
≥ 50 yrs	238	(54.6)
***District***		
Dordogne	57	(13.1)
Gironde	172	(39.4)
Landes	60	(13.8)
Lot-et-Garonne	38	(8.7)
Pyrénées-Atlantiques	94	(21.6)
Unknown	15	(3.4)
**Practice setting**		
Urban	321	(73.6)
Rural	115	(26.4)
**Number of years of medical practice**		
<21 yrs	212	(48.6)
≥ 21 yrs	224	(51.4)
**Working situation**		
Alone	188	(43.1)
Partnership	242	(55.5)
**Weekly working time**		
<55 hours	205	(47.0)
≥ 55 hours	231	(53.0)
**Training in oncology**		
Yes	64	(14.7)
No	355	(81.4)
**Training in geriatrics**		
Yes	120	(27.5)
No	308	(70.6)
**Percentage of patients over 70 yrs**		
<10%	36	(8.3)
10 to 15%	146	(33.5)
15 to 20%	115	(26.4)
20 to 30%	89	(20.4)
>30%	16	(3.7)
**Chronological age at which considered as "elderly"**		
≥ 60 yrs	3	(0.7)
≥ 65 yrs	29	(6.6)
≥ 70 yrs	147	(33.7)
≥ 75 yrs	169	(38.8)
≥ 80 yrs	86	(19.7)

Nearly 50% of the GPs considered that it was difficult to refer a patient to a team of cancer specialists. However, almost 90% considered that cancer specialists readily agreed to take on elderly patients with cancer. Thirty percent of the GPs had suggestions for improving the care of elderly cancer patients, mainly relating to care provision in the home, multidisciplinary care, integration of the GP into decision-making procedures, psychological care of the patients and their families, and the improvement of palliative care.

### Referral decisions: general and clinical case vignettes

Just over half of GPs reported that in general, they "always" referred elderly cancer patients to a cancer team for early cancer cases (230): 53.2% (CI 95%[48.4-58.0]) and just under half "always" referred advanced cancer cases (202): 46% (CI 95%[42.1-51.7]) (p < 0.0001).

Patient-related factors were selected as influencing general referral decisions, such as the degree of mental and physical autonomy, unsuitability of undertaking invasive investigations, presence or absence of serious comorbidity, wish of the family if present, and/or wish or reluctance of the patient. Several, disease-related factors were also selected by a majority of GPs, such as, the severity of cancer symptoms, expected side effects or tolerance towards treatment, and one organisational factor: whether they were in the habit of collaborating with specialist cancer teams (table 2).

**Table 2:** Elements influencing the decision by general practitioners (436) to refer an elderly cancer patient

Elements influencing GPs	Cancer (non specified)		Prostate cancer		Sigmoid colon cancer	
			
	N	(%)	N	(%)	N	(%)
Wish or reluctance of patient (P*)	364	(83.5)^*†*^	158	(36.2)^*†*^	66	(15.2)
Presence of and/or wish of family (P)	348	(79.8) ^*†*^	21	(4.8)	107	(24.6)
Presence or absence of serious comorbidity (P)	347	(79.5)	79	(18.1)	115	(26.4)
Invasive investigations unsuitable (P)	334	(76.6)	52	(11.9)	33	(7.6)
Degree of mental and physical autonomy (P)	328	(75.2)	126	(28.9)	93	(21.4)
Patient's psychological state (P)	307	(70.0)	108	(24.8)	63	(14.5)
Short patient life expectancy (P)	300	(68.4)	32	(7.3)	24	(5.5)
Awareness of diagnosis by patient (P)	254	(58.1)	36	(8.3)	36	(8.3)
Real age of patient (P)	243	(56.3)	113	(25.9)	81	(18.6)
Patient's financial resources (P)	82	(18.4)	1	(0.2)	0	(0.0)
Side effects and tolerance towards treatment (expected) (T^*‡*^)	348	(79.8) ^*†*^	24	(5.5)	14	(3.2)
Seriousness of cancer symptoms (T)	345	(79.1)	131	(30.0) ^*†*^	143	(32.8) ^*†*^
Stage of the disease (T)	314	(71.9)	90	(20.6)	192	(44.1) ^*†*^
Anatomical localisation of the cancer (T)	294	(67.4)	172	(39.4) ^*†*^	151	(34.7) ^*†*^
Presence of good clinical practice guidelines(T)	293	(67.2)	34	(7.8)	23	(5.3)
In the habit of collaborating with specialised cancer teams (O^§^)	337	(77.2)	46	(10.6)	58	(13.3)
Time lapse before care is instated (O)	314	(71.9)	27	(6.2)	25	(5.7)
Organisational difficulties in providing care (O)	222	(50.2)	34	(7.8)	31	(7.1)
Other	55	(12.8)	32	(7.3)	19	(4.4)

*: Patient-related factors (P); †: the three elements mainly influencing the GPs in the three different situations; ‡: Tumour-related factors (T); §: organisational factors (O)

For the two clinical case vignettes concerning a prostate cancer and a sigmoid colon cancer, the majority of GPs reported that they would refer the patient to a team of specialists, approximately 90% and 95% respectively.

The response patterns were different indicating that the patient factors were most commonly selected as influencing general referral decisions, whereas tumour-related factors were most commonly selected for specific cases.

### Independent factors associated with the decision to "always" refer

In the general situation, independent factors associated with the decision to "always" refer an elderly patient with early versus advanced cancer were studied separately. For early cancer, GPs reporting the influence of organisational difficulties relating to care provision (OR = 0.35 95%CI[0.24-0.56], p < 0.0001) and of the anatomical localisation of the disease (OR = 0.58 95%CI[0.37-0.92], p = 0.02) were less likely to refer their patients to a specialist team. No GP characteristic was associated with this decision. For advanced cancer, GPs that had attended training courses in oncology more frequently referred their patients (OR = 1.85 95%CI[1.01-3.38], p = 0.04), whereas no other individual GP characteristic was associated with this decision. Three subgroups of GPs were identified as being less likely to refer their patients. These were GPs that reported being influenced by patient age (OR = 0.55 95%CI[0.35-0.86], p = 0.009), organisational difficulties in providing care (OR = 0.60 95%CI[0.39-0.92], p = 0.02) and the stage of the disease (OR = 0.43 95%CI[0.25-0.71], p = 0.001) (table 3).

**Table 3:** Determinants of the decision to refer elderly patients at early and advanced stage (multivariate analyse)

	β	SE	OR	[CI 95%]	p
**"Early disease" model (417 GPs)**					
**Elements influencing GPs**					
Organisational difficulties (care provision) (ref: not influenced)	-0.99	0.21	0.37	[0.24-0.56]	<0.0001
Anatomical localisation of the cancer (ref: not influenced)	-0.53	0.23	0.58	[0.37-0.92]	0.02
R-Squared = 0.08; Chi-Squared Hosmer and Lemeshow = 0.02 (p = 0.99)					
**"Advanced disease" model (397 GPs)**					
**GP characteristics**					
Training in oncology (ref: not influenced)	0.61	0.31	1.85	[1.01-3.38]	0.046
**Elements influencing GPs**					
Chronological age of patient (ref: not influenced)	-0.59	0.23	0.55	[0.35-0.86]	0.009
Organisational difficulties (care provision) (ref: not influenced)	-0.51	0.22	0.60	[0.39-0.92]	0.02
Disease stage (ref: not influenced)	-0.85	0.26	0.43	[0.25-0.71]	0.001
R-Squared = 0.11; Chi-Squared Hosmer and Lemeshow = 4.31 (p = 0.63)					

## Discussion

In our study, approximately half of the GPs declared that they always refer elderly cancer patients to a cancer team (this was slightly more frequent for early stages than for advanced disease). More than three quarters of referring and non-referring GPs reported being influenced by the five following patients-linked factors: 1) wish or reluctance on the part of the patient; 2) wish of the family if present; 3) presence or absence of serious comorbidity; 4) unsuitability of conducting invasive investigations; and 5) the degree of mental and physical autonomy. Three quarters or more of the GPs were influenced by two disease-linked factors, the seriousness of the cancer symptoms and expected side effects and tolerance of treatment. Finally, being used to collaborating with specialist cancer teams was the only organisational element reported to be influential by more than 75% of the GPs. With regard to the two specific cancer cases presented in clinical case vignettes, the GPs did not have the same approach for the two patients, nor when compared to the general referral situation. Confronted with a case of prostate cancer, the GPs tended to refer the patient to a specialist (75%), whereas when faced with a sigmoid colon cancer, 45% of GPs reported that they would refer to a specialist and 40% to an oncologist. For both these types of cancer, the seriousness of the symptoms appears to influence GPs the most. Regardless of the stage of the cancer, organisation difficulties were an independent factor influencing the GP's decision whether or not to refer elderly patients.

In studies reported in the literature and conducted in other regions in France and in Canada [10,12], higher referral rates of elderly patients presenting with cancer have been reported according to patient's age [12] and to stages of the disease [10]. Our referral rates are slightly lower in the general situation, but we have similar high referral rates for both clinical vignettes. Overall, slightly more GPs declared that they "always refer" for early stages than for advanced disease which is similar to results previously reported [10]. In Canada, as in France today, patients encountering a health problem consult in the first instance the family practitioner (GP) who decides if referral to a specialist is required. It is possible that in the present study the figure for referral is underestimated on account of the absence in France for a clear definition of a "team of cancer specialists", so that the GPs may not have included specialists practising oncology under the term.

In terms of the factors influencing GPs' referral decisions, we found the same factors to be cited in majority (patient's wishes and tumour-related factors) as observed previously [10,12]. When independent factors associated with the decision to "always" refer an elderly patient to a cancer team for early stages of the disease are considered, GPs reporting being influenced by the anatomical localisation of the cancer reported referred their patients less often. In the limited literature available, the anatomical localisation has never been documented as a factor influencing GPs' decisions. However, this factor seems likely to play a part in decisions on the way a cancer patient is to be cared for, and this aspect was confirmed in this study. In particular, our results show that the GPs did not refer patients in the same way in the general situation (responses to questionnaire Section B) and in the two clinical situations proposed. Another decisional factor found, irrespective of disease extension, was the difficulty involved in organising care. This factor is also found in another two studies conducted on a sample of GPs where organisational difficulties were found to influence decisions [10,12]. This has also been observed in studies conducted among elderly breast and colon cancer patients where the patients reported preferring to receive care close to their homes [13,14]. Indeed, cancer care that is often complex and requires frequent consultations may be difficult to organise for elderly patients living at a distance from the specialised cancer centres. In this context, the GP may choose to refer a patient to a closer facility such as, a geriatric or medical ward [6]. Despite this, the practice setting (urban or rural) was not found as a significant factor in any of the models in the present study. For advanced cancer, GPs reporting that they "did not always refer" their patients were those not having attended courses in oncology, and those influenced by the chronological age of their patients, the stage of advancement of the disease, or organisational difficulties for care. For this group, age itself was a determining factor of the referral decision. This association has not been documented in the literature up until now. This difference may not have been observed in the Canadian study as it was performed some time ago and in a different health system [10]. In the French study, the stage was not taken into account when studying referral decisions [12]. Oncology training was found to increase referral rates in our study, but we did not observe an association with geriatric training that has previously been reported in the literature as decreasing referral rates [10,12].

The GPs did not refer their patients to the same specialists in the two clinical case vignettes. For prostate cancer patients, GPs referred their patients to a urological specialist. For patients with colon cancer, GPs referred their patients to a gastro-enterologist or to an oncologist. Specialists' attitudes towards oncogeriatrics and established collaboration relationships can have an important impact on the initial management of patients [15].

Finally, there are two main limitations to this study to keep in mind when interpreting results. The first relates to the type of survey, with this being a postal survey conducted among GPs in the South-West region of France, Aquitaine. After two reminders, an acceptable response rate for this type of study was obtained (30%). At first regard, this may seem low and indicate a selection bias, but since only 50% of graduated doctors are thought to be in general practice in France [16], probably only 50% of the GPs listed in the regional database we used to generate our participant list were actually concerned by the study. Kurtz et al [12] showed a higher GP response rate but this difference can be explained by the different types of questionnaire (they did not present clinical case vignettes) and they employed a more direct regional communication method to obtain GP responses. In our group of respondents, there were slightly more males and GPs with a rural practice than in the regional GP database. An explanation could be that these GPs see more elderly patients so they were more interested in participating in the study.

The second limitation concerns the fact that the clinical cases vignettes covered two specific disease localisations and the GPs were probably influenced by the prognosis for these specific cancers.

## Conclusions

Overall, our results indicate that it is still necessary to raise awareness among physicians so that all patients, regardless of age, stage of disease or anatomical localisation of the cancer, be seen by a team of oncologists in order for a decision suited to the patient to be reached.

Three specific elements influencing GPs' decisions for the referral of elderly patients with cancer to a cancer team should be highlighted in our results as the focus for future research or interventions. The organisational difficulties encountered by GPs highlight the need for further studies to comprehend the nature of these difficulties and to suggest solutions suited to the local setting. Another factor identified concerns the lack of training in oncology that should be developed in the future. The role of specialist physicians is likewise important in the promotion of oncogeriatrics among GPs.

## List of abbreviations used

GP: General Practitioners; CI: Confidence Interval; sd: standard deviation

## Competing interests

The authors declare that they have no competing interests.

## Authors' contributions

FD: conceived of the study, participated in its design and coordination, performed the statistical analysis and drafted the manuscript; EM: helped with acquisition of data, interpretation of data and revising the manuscript; MF: participated in interpretation of data and revising the manuscript; MR: participated in interpretation of data and revising the manuscript; JLD: participated in interpretation of data and revising the manuscript; PM: participated in interpretation of data and revising the manuscript; PS: participated in interpretation of data and revising the manuscript; AJS: participated in interpretation of data and revising the manuscript; SMP: conceived of the study, participated in its design and coordination and revising statistical analysis and the manuscript. All authors read and approved the final draft.

## Pre-publication history

The pre-publication history for this paper can be accessed here:

http://www.biomedcentral.com/1471-2407/11/5/prepub

## Supplementary Material

Additional file 1 **questionnaire for the study of factors that influence general practitioners in the referral of elderly cancer patients**. The final version comprises four sections and two clinical case vignettes. Section A concerns the GP's elderly patients overall (GP's perception of the age at which a person is "elderly", percentage of elderly people among patients overall). Section B concerns referral to a specialised oncology team for the GPs' elderly patients with cancer, in two categories: early or advanced cancer. Section C explored any training received by the GPs in oncology or geriatrics, and the perception they had of the way elderly cancer patients are cared for in France. Section D recorded the characteristics of the responding GPs. Finally, two clinical case vignettes were provided to investigate the GP's attitude in a clinical situation: one case of prostate cancer and one sigmoid colon cancer case. Click here for file
