# Supplementary material for: Factors influencing general practitioners in the referral of elderly cancer patients
Source: BMC Cancer. 2011 Jan 6;11:5. doi: 10.1186/1471-2407-11-5 (PMC3024300; doi:10.1186/1471-2407-11-5)
Supplement: Additional file 1 — questionnaire for the study of factors that influence general practitioners in the referral of elderly cancer patients. The final version comprises four sections and two clinical case vignettes. Section A concerns the GP's elderly patients overall (GP's perception of the age at which a person is "elderly", percentage of elderly people among patients overall). Section B concerns referral to a specialised oncology team for the GPs' elderly patients with cancer, in two categories: early or advanced cancer. Section C explored any training received by the GPs in oncology or geriatrics, and the perception they had of the way elderly cancer patients are cared for in France. Section D recorded the characteristics of the responding GPs. Finally, two clinical case vignettes were provided to investigate the GP's attitude in a clinical situation: one case of prostate cancer and one sigmoid colon cancer case. [file 1471-2407-11-5-S1.DOC]

Referral of elderly cancer patients

**Surname:** ________________________ **First name:** ____________________

(The information collected will be anonymous at the moment data entry. Providing your name allows us to send you study results)

**Age:** _ _ years **Sex:** 🞎 M 🞎 F **Department:** _ _

## Elderly patients in your practice?

## *(Please tick the boxes of your choice)*

1. At what chronological age (as on official documents) would you consider a patient to be “elderly”? *(1 response)*

🞎 ≥ 60 years old

🞎 ≥ 65 years old

🞎 ≥ 70 years old

🞎 ≥ 75 years old

🞎 ≥ 80 years old

1. What percentage of patients, in your practice, are aged 70 years or older? *(Data available in your « Relevés Individuels d’Activité et de Prescriptions (RIAP) ») (1 response)*

🞎 <10%

🞎 10 à 20%

🞎 20 à 30%

🞎 30 à 50%

🞎 > 50 %

## Referral to a specialised cancer team for elderly patients?

1. Would you refer your **patients aged 70 years or older**, with a presumed diagnosis of an **early stage** of cancer to a specialized cancer team? *(1 response)*

🞎 Very rarely (<10%)

🞎 Rarely (10-25%)

🞎 Sometimes (25-50%)

🞎 Often (50-75%)

🞎 Always (>75%)

1. Would you refer your **patients aged 70 years or older**, with a presumed diagnosis of an **advanced stage** of cancer to a specialized cancer team? *(1 response)*

🞎 Very rarely (<10%)

🞎 Rarely (10-25%)

🞎 Sometimes (25-50%)

🞎 Often (50-75%)

- Always (>75%)

1. What factors influence your decision to refer your elderly patients with cancer to a **specialized cancer team**? *(For each factor circle your response: 1 yes, 2 somewhat, 3 not at all)*

| **1 2 3** | Chronological age of patient | **1 2 3** | Invasive investigations unsuitable |
| --- | --- | --- | --- |
| **1 2 3** | Presence or absence of serious comorbidity | **1 2 3** | Anatomical localization of the cancer |
| **1 2 3** | Patient’s psychological state | **1 2 3** | Stage of the disease |
| **1 2 3** | Degree of mental and physical autonomy | **1 2 3** | Seriousness of cancer symptoms |
| **1 2 3** | Awareness of diagnosis by patient | **1 2 3** | Side effects and tolerance towards treatment (expected) |
| **1 2 3** | Presence of and/or wish of family | **1 2 3** | Presence of good clinical practices guidelines |
| **1 2 3** | Patient’s financial resources | **1 2 3** | In the habit of collaborating with specialized cancer teams |
| **1 2 3** | Organisational difficulties in providing care | **1 2 3** | Time lapse before care is instated |
| **1 2 3** | Wish or reluctance of patients | **1 2 3** | Others, specify: |
| **1 2 3** | Short patient life expectancy |  |  |

1. What would influence you more in deciding whether or not to refer an elderly patient with cancer? *(1 response)*

- Age
- Well-being

## Organization for care of elderly cancer patients

1. Do you have training in :

**Geriatrics**: Yes 🞎 No 🞎

If yes, what type: Complementary (DU, DIU, …) 🞎 What year ? _____

Continuing education 🞎 Final year: _____

**Oncology**: Yes 🞎 No 🞎

If yes, what type: Complementary (DU, DIU, …) 🞎 What year ? _____

Continuing education 🞎 Final year: _____

1. Do you find it easy or difficult to refer any patients to a specialized cancer team?

- Easy
- Sometimes difficult
- Difficult

1. Do you find specialized cancer teams accept your elderly patients easily?

- Yes
- Mostly yes
- Mostly no
- No

1. Do you think that in France there are appropriate courses (whatever the content) to help general practitioners to manage elderly cancer patients’ care:

- Yes
- No

If no, what are your suggestions to improve these courses?

1. Do you have suggestions to improve the care of elderly cancer patients?

- Yes
- No

If yes, what are these?

## Your characteristics

1. How many years have you been practicing general medicine?: _ _ years
2. Practice setting : urban 🞎 rural 🞎
3. How many hours per week do you work? (self-employment activity) : _ _ h
4. Work organization: alone : 🞎 in partnership : 🞎

## « Clinical case »

## « Clinical case » n°1

1. During a routine renewal of prescription visit for well-managed high blood pressure and memory trouble, Mr L., 80 years old, asks you at the last moment for a treatment for back pain that wakes him during the night. He believes that the back pain is coming from the difficulty he is experiencing passing urine which has been getting worse for some time and means that he needs to force to urinate. He is a widower and has one child who lives 200km away.

The clinical examination (including neurological) is normal but he refuses the rectal inspection “it’s nothing, it will go away, it’s just part of getting old”. In the end, you prescribe a blood sample, a Cytobacterial urine examinatio*n,* a x-ray of the lumbar spine and suggest he comes back early next week. The PSA dose is 350; the CBUE and x-ray are without symptoms.

You see him again as planned and he says that he is less troubled now that he has medication. You explain to him the possibility of cancer. He still refuses a rectal inspection.

16.1/ What do you do after this assessment? *(1 response)*

- Adjustment of symptomatic treatment and reassessment
- You propose an urologist consultation
- You refer to an oncologist
- Other, specify :

16.2/ What are three factors that most influence your decision? (Tick three boxes corresponding to your choice*)*

| 🞎 | Chronological age of patient | 🞎 | Invasive investigations unsuitable |
| --- | --- | --- | --- |
| 🞎 | Presence or absence of serious comorbidity | 🞎 | Anatomical localization of the cancer |
| 🞎 | Patient’s psychological state | 🞎 | Stage of the disease |
| 🞎 | Degree of mental and physical autonomy | 🞎 | Seriousness of cancer symptoms |
| 🞎 | Awareness of diagnosis by patient | 🞎 | Side effects and tolerance towards treatment (expected) |
| 🞎 | Presence of and/or wish of family | 🞎 | Presence of good clinical practices guidelines |
| 🞎 | Patient’s financial resources | 🞎 | In the habit of collaborating with specialized cancer teams |
| 🞎 | Organizational difficulties in providing care | 🞎 | Time lapse before care is instated |
| 🞎 | Wish or reluctance of patients | 🞎 | Others, specify: |
| 🞎 | Short patient life expectancy |  |  |
|  |  |  |  |

## « Clinical case » n°2

1. **You receive a blind man aged 73 years who has been brought to you by his sister in law due to a general deterioration, abdominal pain and recent constipation.**

**He spends his day seated in an armchair, does not speak a lot and does not eat a lot. She has had to force him to come to see you. She looks after him and his brother.**

**His medical history shows a heart attack with double coronary bypass 5 years ago.**

**During your clinical examination, you can feel a palpable mass in the left iliac fossa and a hepatomegaly. No fecaloma is found during rectal inspection.**

**You prescribe an abdominal ultra-sound (widening of the sigmoid associated with 2 hepatic nodules) and a blood sample (haemoglobins 10g/dl, high CEA) and a second visit once the results are received.**

**The patient and the anxious sister in law ask you what they should do.**

17.1/ What do you do after this assessment? *(1 response)*

- Adjustment of symptomatic treatment and reassessment
- You propose a gastroenterologist consultation
- You refer to an oncologist
- Others, specify :

17.2/ What are the three factors that most influence your decision? (Tick three boxes corresponding your choice*)*

| 🞎 | Chronological age of patient | 🞎 | Invasive investigations unsuitable |
| --- | --- | --- | --- |
| 🞎 | Presence or absence of serious comorbidity | 🞎 | Anatomical localization of the cancer |
| 🞎 | Patient’s psychological state | 🞎 | Stage of the disease |
| 🞎 | Degree of mental and physical autonomy | 🞎 | Seriousness of cancer symptoms |
| 🞎 | Awareness of diagnosis by patient | 🞎 | Side effects and tolerance towards treatment (expected) |
| 🞎 | Presence of and/or wish of family | 🞎 | Presence of good clinical practices guidelines |
| 🞎 | Patient’s financial resources | 🞎 | In the habit of collaborating with specialized cancer teams |
| 🞎 | Organisational difficulties in providing care | 🞎 | Time lapse before care is instated |
| 🞎 | Wish or reluctance of patients | 🞎 | Others, specify: |
| 🞎 | Short patient life expectancy |  |  |
|  |  |  |  |

## Do you have any remarks on this questionnaire or on the subject of « elderly patients and cancer »?

***Thank you for sending this questionnaire with a prepaid envelope to: Unité Pilote de Coordination en Oncogériatrie, 229 cours de l’Argonne, 33076 Bordeaux cedex***
